# Paternal violent criminality and preterm birth: a Swedish national cohort study

**DOI:** 10.1186/s12884-020-02964-2

**Published:** 2020-05-19

**Authors:** Can Liu, Niklas Långström, Cecilia Ekéus, Thomas Frisell, Sven Cnattingius, Anders Hjern

**Affiliations:** 1 Centre for Health Equity Studies (CHESS), Karolinska Institutet/Stockholm University, 106 91 Stockholm, Sweden; 2 Clinical Epidemiology, Department of Medicine, Karolinska Institutet, 171 77 Stockholm, Sweden; 3 Department of Neuroscience, Uppsala University, Box 256, 751 05 Uppsala, Sweden; 4 Department of Medical Epidemiology and Biostatistics, Karolinska Institutet, 171 77 Stockholm, Sweden; 5 Division of Reproductive Health, Department of Women’s and Children’s Health (KBH), 171 77 Stockholm, Sweden

**Keywords:** Violent crime, Psychosocial stress, Father, Preterm birth

## Abstract

**Background:**

Fathers may affect expectant mothers’ daily living situations, which in turn might influence pregnancy outcomes. We investigated the association between paternal violent criminality and risk of preterm birth (≤36 weeks).

**Methods:**

We conducted a register-based study with all live singleton births in the Swedish Medical Birth Register from 1992 to 2012, linked with records of paternal violent crime convictions from the National Crime Register from 1973 to 2012.

**Results:**

Paternal violent criminality was associated with increased risk of preterm birth and lower gestational age. The association was especially pronounced among infants of reoffenders: men convicted of three or more violent crimes (adjusted odds ratio [aOR] 1.23 [95% CI 1.17, 1.29]). Maternal half sibling-comparisons, an analytic approach controlling for maternal factors stable across pregnancies, also suggested increased risk of preterm birth and lower gestational age when exposed to a violently reoffending father compared to a father without violent criminal convictions (aOR 1.30 [0.99, 1.72], adjusted mean difference − 1.07 [− 1.78, − 0.36]).

**Conclusions:**

Persistent paternal violent criminality was associated with increased risk of preterm birth, even after controlling for maternal characteristics that did not change between pregnancies.

## Background

Preterm birth is the leading cause of infant mortality worldwide. Although there are several known risk factors for preterm birth, the aetiology for most preterm births remains elusive [1]. Maternal psychosocial stress is one suggested mechanism, particularly for spontaneous preterm birth [2–5].

The expectant father is an integral part of the psychosocial and physical environment of the pregnant woman, and may thereby influence the pregnancy [6]. Partner engagement and support was associated with more proactive antenatal care attendance and less perinatal distress in clinic-based studies [7, 8]. A national population study suggested that prenatal paternal depression may increase preterm birth risk [9]. However, empirical studies that pay attention to partner relationships and father’s involvement before childbirth remain scarce [2].

Violence is a severe global public health problem [10]. Having an aggressive partner may not only increase the risk of domestic violence victimization, but can also be a source of severe stress. However, estimating the health consequences of violence is hindered by data availability [10], and convicted violent crime is only the tip of the iceberg. Compared to men without conviction records, those convicted of violent crime, particularly reoffenders, are more likely to behave violently in everyday interaction with their partners. Beyond being a strong proxy for violent behaviour, conviction status is also a potential social determinant of offender health, which might affect the entire family [11] as suggested by studies of families of incarcerated offenders [12–14].

By cross-linking national Swedish registers, we explored whether a father’s conviction status increases the risk of preterm birth and shortens gestational age. To test whether maternal characteristics partly explained possible associations, we also compared maternal half-siblings whose fathers differed by conviction status.

## Methods

Data from longitudinal national medical and social registers in Sweden were linked using the person-unique national identification numbers, assigned to each Swedish resident. After data linkage, personal identification numbers were replaced with non-traceable distinctive identifiers to secure anonymity.

### Study population

**Fig. 1:**
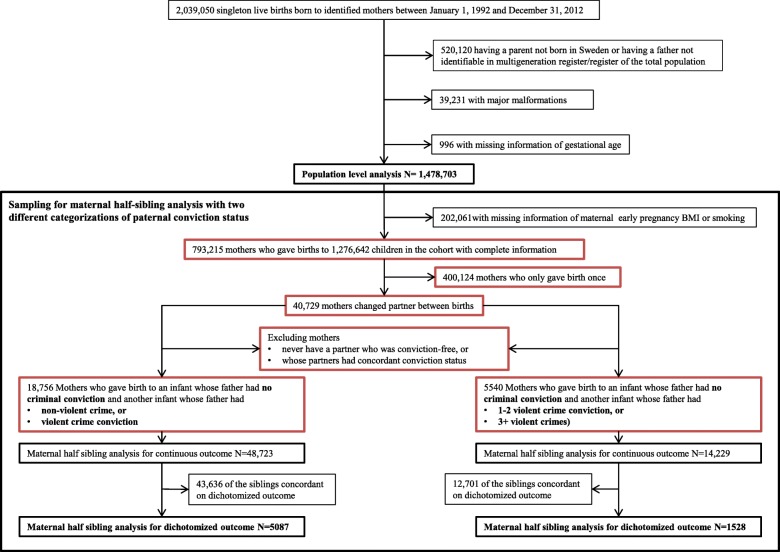
Sampling of the population and sibling comparison

All live singleton births from January 1, 1992 to December 31, 2012 and their mothers were identified from the Swedish Medical Birth Register (SMBR) (*n* = 2,039,050). Fathers were identified by linkage to the Multigeneration Register, which record biological and adoptive parents of all Swedish residents. Subjects with unidentified or foreign-born parents were excluded from the study population (*n* = 520,120), due to different durations of living in Sweden, related to the likelihood of having criminal record. After excluding births with non-minor malformations (*n* = 39,231) and those missing gestational age data (*n* = 996), the study population consisted of 1,478,703 singleton births. Additional sampling for maternal half-sibling comparisons is described in Fig. 1.

Perinatal and psychiatric diagnoses were coded in accordance with the International Classification of Disease (ICD). The ninth version (ICD-9) was used up until 1996, and the tenth version (ICD-10) was used thereafter.

### Exposure to father ever convicted of violent crime

Information on paternal criminal conviction status was derived from the Crime Register and categorized as having no criminal conviction, having a violent criminal conviction, and having another non-violent criminal conviction (see Supplementary Table 1 for the corresponding penal codes). The total number of violent crime convictions was calculated and dichotomised into non-reoffenders and reoffenders, defined as having one to two versus three or more violent offences. We included those convicted after the birth in the exposed group, considering the low risk of reverse causation and the explanatory factors being prevailing long before the conviction.

### Outcome variables

Gestational age in days was collected from the SMBR based on ultrasound-corrected estimation. Preterm birth was defined as a gestational age of 36 completed weeks or less, and was further characterized into very preterm (22–31 weeks) and moderately preterm (32–36 weeks) birth. We defined a spontaneous preterm birth as having a preterm birth with spontaneous onset of delivery or a maternal diagnosis of preterm rupture of the membranes (ICD-9 code 658.1; ICD-10 code O42). Medically indicated preterm birth was defined as having an induced onset of delivery, including a caesarean section.

### Covariates

We collected information on maternal age, parity, height, smoking status, body weight, country of birth, family situation at first antenatal care visit, and year of delivery from the SMBR. Maternal criminal conviction status was also obtained from the Crime Register and categorized as no criminal conviction, a violent criminal conviction and a non-violent criminal conviction, respectively. Data on dispensable household income per person the calendar year before childbirth, paternal age, maternal and paternal education until the year of childbirth, were collected from the Swedish Income and Enumeration Survey, held by Statistics Sweden. We did not collect data on neighbourhood context, as previous research suggests that neighbourhood context does not directly affect criminality or preterm birth in the Swedish context [15, 16].

In the Swedish judicial system, even severe mental illness does not exclude criminal sentencing. Thus, some of the crimes were conducted under influence of psychiatric disorder. Paternal and maternal psychiatric diagnoses (ICD-9 codes 293–319, excluding 303–305.0; ICD-10 codes F00-F99, excluding F11-F19), alcohol use (ICD-9 codes 291, 303, 305.0, 357.5, 425.5, 535.3, 571.0–571.3; ICD-10 codes F10, K70, G621, I426, K292), and drug use (ICD-9 codes 292, 304, 965.0, 968.5, 969.6, 969.7; ICD-10 codes F11, F12, F14, F16, F19, Z503, Z71.5, Z72.2, O35.5, P04.4, T40, T43.6) diagnoses were collected from the National Patient Register covering all inpatient admissions (recorded from 1973 onwards) and non-primary outpatient care (information available from 2001 onwards).

### Statistical analysis

In population analysis, we first compared births exposed to paternal non-violent and violent criminal convictions to births fathered by men without criminal convictions. Secondly, we compared births fathered by those convicted of 1–2 and 3 or more violent offences, respectively, to those without criminal convictions. In these two analyses, we used logistic regression models with cluster robust estimation of standard errors, adjusting for calendar year of birth, parity, maternal and paternal age (in quadratic forms), maternal and paternal education. We also performed linear regression models of the same variables to estimate the mean differences in gestational age (βs) between the groups. Thirdly, we compared risks of spontaneous or medically indicated preterm birth and risks of very or moderately preterm birth using multinomial logistic regression models.

Sibling comparisons between the exposed infant and his/her unexposed maternal half-sibling allowed controlling for stable maternal confounders. Fixed effect linear (STATA command xtreg, fe) and logistic regression (STATA command xtlog, fe) modelling was performed to estimate βs and ORs of preterm birth of exposed infant in comparison to his/her unexposed maternal half-sibling, whose father had no criminal conviction status. Cluster robust estimation of standard errors was used in fixed effect models to account for independence between maternal half-siblings, with Huber/White/sandwich estimator and bootstrap estimator being used in fixed linear and logistic regression, respectively. In addition to variables adjusted in population analysis, further adjustment was made for pregnancy-specific covariates of quintiles of household income per person, maternal body mass index (BMI) and smoking status in early pregnancy.

Stratified analysis was conducted to explore potential modification of cohabitation situation on paternal violent criminality. We also tested for multiplicative interactions between paternal violent criminal conviction status (1–2 and 3 or more vs. no criminal conviction) and family situation.

Data management was performed using SAS 9.4 (SAS institute, Cary, NC). All statistical analyses were performed using STATA.

## Results

Table 1 shows that about 8% of all births were fathered by men convicted for one or more violent crimes. Factors associated with being exposed to a father convicted of violent crime included relatively young parental age (below 25 years), low education, low household income per person, not cohabiting parents, maternal smoking during pregnancy, fathers or mothers ever having psychiatric or substance misuse diagnoses, and if mothers were convicted.

**Table 1:** Proportion with convicted father by characteristics of birth (*N* = 1,478,703)

	Conviction status of the father					
	No crime conviction		Non-violent crime		Violent crime	
	n	(%)	n	(%)	n	(%)
Parity						
1	414,872	(44.5)	179,329	(41.5)	50,231	(43.5)
2–3	479,359	(51.5)	225,703	(52.3)	55,386	(48.0)
4 or higher	37,399	(4.0)	26,685	(6.2)	9739	(8.4)
Maternal age						
11–19 years	8818	(1.0)	8071	(1.9)	6306	(5.5)
20–24 years	109,621	(11.8)	65,636	(15.2)	28,130	(24.4)
25–29 years	316,388	(34)	143,304	(33.2)	37,525	(32.5)
30–34 years	333,008	(35.7)	139,043	(32.2)	28,061	(24.3)
≥ 35 years	163,795	(17.6)	75,663	(17.5)	15,334	(13.3)
Paternal age						
11–19 years	2130	(0.2)	1632	(0.4)	1527	(1.3)
20–24 years	47,613	(5.1)	26,316	(6.1)	14,074	(12.2)
25–29 years	226,876	(24.4)	100,043	(23.2)	30,947	(26.8)
30–34 years	348,732	(37.4)	145,557	(33.7)	33,415	(29.0)
≥ 35 years	306,279	(32.9)	158,169	(36.6)	35,393	(30.7)
Maternal education						
Compulsory school ≤9 years	57,410	(6.2)	55,070	(12.8)	30,537	(26.5)
Secondary school	189,962	(20.4)	124,545	(28.9)	35,503	(30.8)
University < 3 years	386,587	(41.5)	170,670	(39.5)	39,261	(34.0)
University ≥3 years	297,671	(32.0)	81,432	(18.9)	10,055	(8.7)
Paternal education						
Compulsory school ≤9 years	69,536	(7.5)	72,805	(16.9)	39,013	(33.8)
Secondary school	244,316	(26.2)	165,265	(38.3)	46,473	(40.3)
University < 3 years	382,884	(41.1)	142,633	(33.0)	25,407	(22.0)
University ≥3 years	234,894	(25.2)	51,014	(11.8)	4463	(3.9)
Quintile of household income per person						
Below 20 percentile	85,323	(9.2)	66,065	(15.3)	32,261	(28.0)
20–40 percentile	167,265	(18.0)	90,014	(20.9)	29,265	(25.4)
40–60 percentile	208,938	(22.4)	94,213	(21.8)	23,972	(20.8)
60–80 percentile	229,877	(24.7)	92,402	(21.4)	18,459	(16.0)
Above 80 percentile	240,097	(25.8)	88,934	(20.6)	11,323	(9.8)
Missing	130	(0)	89	(0)	76	(0.1)
Family situation						
Co-habiting with the father	860,329	(92.4)	386,555	(89.5)	95,127	(82.5)
Not co-habiting with the father	21,880	(2.4)	20,120	(4.7)	13,636	(11.8)
Missing	49,421	(5.3)	25,042	(5.8)	6593	(5.7)
Maternal smoking status						
No smoking	824,035	(88.5)	344,162	(79.7)	75,964	(65.9)
1–9 cigarettes/d	45,650	(4.9)	44,509	(10.3)	21,545	(18.7)
10+ cigarettes/d	17,434	(1.9)	21,584	(5.0)	12,218	(10.6)
Missing	44,511	(4.8)	21,462	(5.0)	5629	(4.9)
Maternal BMI						
Underweight (< 18.5)	17,258	(1.9)	9169	(2.1)	3366	(2.9)
Normal weight (18.5 to 25)	534,722	(57.4)	239,557	(55.5)	62,497	(54.2)
Overweight (25 to 30)	191,187	(20.5)	87,312	(20.2)	23,320	(20.2)
Obese Class I (30 to 35)	56,443	(6.1)	27,494	(6.4)	7925	(6.9)
Obese Class II (≥35)	21,753	(2.3)	11,082	(2.6)	3218	(2.8)
Missing	110,267	(11.8)	57,103	(13.2)	15,030	(13.0)
Paternal psychiatric diagnosis						
At least once	51,426	(5.5)	44,653	(10.3)	30,628	(26.6)
Maternal psychiatric diagnosis						
At least once	104,608	(11.2)	64,186	(14.9)	27,652	(24.0)
Paternal alcohol use disorder						
At least once	6480	(0.7)	15,001	(3.5)	15,292	(13.3)
Maternal alcohol use disorder						
At least once	5717	(0.6)	6125	(1.4)	4276	(3.7)
Paternal drug use disorder						
At least once	1474	(0.2)	6975	(1.6)	12,793	(11.1)
Maternal drug use disorder						
At least once	3814	(0.4)	4635	(1.1)	4944	(4.3)
Having a maternal half-sibling						
Yes	62,717	(6.7)	49,391	(11.4)	24,319	(21.1)
Maternal conviction						
No crime conviction	862,801	(92.6)	373,780	(86.6)	85,722	(74.3)
Non-violent crime	63,857	(6.9)	51,986	(12.0)	23,630	(20.5)
Violent crime	4972	(0.5)	5951	(1.4)	6004	(5.2)

### Population analysis

Table 2 indicates that compared with non-exposed infants (i.e., with a father with no criminal conviction), infants whose father had any violent conviction had increased risks of preterm birth (OR 1.11 [95% CI 1.08 to 1.15]) and shortened gestational age (β − 0.41 [− 0.50 to − 0.32].

**Table 2:** Conviction status of the father and odds ratios (ORs) of preterm birth and mean differences of gestational age in days (βs) in population analysis (*N* = 1,478,703) and sibling comparison (*N* = 48,723 for outcome of gestational age, *N* = 5087 for outcome of preterm birth)

	Preterm birth				Gestational age	
	%	(n/N)	OR	(95%CI)	β	(95%CI)
	**Population analysis**					
No Criminal Conviction	4.5	(41,794/931,630)	1		0	
Convicted of Non-violent Crime	4.8	(20,634/431,717)	**1.02**	(1.00, 1.04)	−0.05	(− 0.10, 0.00)
Convicted of Violent Crime	5.6	(6450/115,356)	**1.11**	(1.08, 1.15)	**−0.41**	(− 0.50, − 0.32)
	**Sibling comparison**					
No Criminal Conviction	38.3	(964/2516)	1		0	
Convicted of Non-violent Crime	39.5	(709/1795)	1.00	(0.85, 1.17)	−0.01	(−0.23, 0.22)
Convicted of Violent Crime	40.7	(316/776)	1.03	(0.85, 1.23)	−0.17	(− 0.51, 0.16)

Note:

Bolded figures are significant at *p* < 0.05

Population analysis adjusted for year of birth, paternal age, maternal age, parity, and paternal education

Sibling comparison adjusted for variables in population analysis and also for quintiles of household income in the calendar year before childbirth, maternal early pregnancy BMI and smoking status

Table 3 shows that Compared to non-exposed infants, infants of reoffending (≥3 violent convictions) fathers had even higher risk of preterm birth (OR 1.23 [1.17 to 1.29]) than infants of fathers with 1–2 violent crime convictions (OR 1.08 [1.04 to 1.12]). Similarly, infants of reoffending fathers had shorter mean gestational age than infants of fathers with 1–2 violent crime convictions (β _≥3_–0.79 [− 0.96 to − 0.62] vs. β _1–2_ -0.29 [− 0.40 to − 0.18], respectively).

**Table 3:** Conviction status of the father and odds ratios (ORs) of preterm birth and mean differences of gestational age in days (βs) in population (*N* = 1,046,986*) and sibling comparison analyses (*N* = 14,229 for outcome of gestational age, *N* = 1528 for outcome of preterm birth)

	Preterm birth				Gestational age	
	%	(n/N)	OR	(95%CI)	β	(95%CI)
	**Population analysis**					
No criminal conviction	4.5	(41,794/931,630)	1		0	
1–2 violent crime convictions	5.3	(4314/81,581)	**1.08**	(1.04, 1.12)	**−0.29**	(−0.40, − 0.18)
3+ violent crime convictions	6.3	(2136/33,775)	**1.23**	(1.17, 1.29)	**−0.79**	(− 0.96, − 0.62)
	**Sibling comparison**					
No criminal conviction	37.9	(290/765)	1		0	
1–2 violent crime convictions	38.1	(180/473)	0.93	(0.71, 1.21)	0.10	(−0.36, 0.56)
3+ violent crime convictions	45.9	(133/290)	1.27	(0.91, 1.78)	**−0.91**	(−1.51, − 0.31)

Note:

Bolded figures are significant at *p* < 0.05

Population analysis adjusted for year of birth, paternal age, maternal age, parity, and paternal education

Sibling comparisons were adjusted for variables in population analysis and also for quintiles of household income in the calendar year before childbirth, maternal early pregnancy BMI, and smoking status

*Excluded 431,717 infants of non-violent fathers from the total study population

Table 4 shows that if the father was convicted three or more times for violent offending, the risks of all four subtypes of preterm birth were increased: spontaneous and medically indicated very (≤31 weeks) and moderately (32–36 weeks) preterm birth. The risk of spontaneous very preterm was especially pronounced (OR 1.49 [1.25 to 1.78]). Infants fathered by men convicted of one to two violent crimes had marginally increased risks of very (OR 1.14 [1.00 to 1.30]) and moderately (OR 1.09 [1.04 to 1.14]) spontaneous preterm birth, but not medically indicated preterm birth. Infants fathered by men convicted of non-violent crimes had no increased risk of either spontaneous or medically indicated preterm birth (Supplementary Table 2).

**Table 4:** Conviction status of the father and odds ratios (ORs) of spontaneous/medically indicated very (≤31 weeks) preterm birth and moderately (32–36 weeks) preterm birth (*N* = 1,046,986*)

	Very preterm birth				Moderately preterm birth			
	%	(n/N)	OR	(95%CI)	%	(n/N)	OR	(95%CI)
	**Medically indicated preterm birth**							
No criminal conviction	0.2	(2149/ 931,630)	1		1.2	(10,959/ 931,630)	1	
1–2 violent crime convictions	0.3	(200/ 81,581)	0.98	(0.84, 1.15)	1.3	(1063/ 81,581)	1.03	(0.96, 1.10)
3+ violent crime convictions	0.3	(108/ 33,775)	1.23	(0.99, 1.51)	1.6	(527/ 33,775)	**1.18**	(1.07, 1.30)
	**Spontaneous preterm birth**							
No criminal conviction	0.3	(2488/ 931,630)	1		2.7	(24,674/ 931,630)	1	
1–2 violent crime convictions	0.4	(293/ 81,581)	**1.14**	(1.00, 1.30)	3.1	(2553/ 81,581)	**1.09**	(1.04, 1.14)
3+ violent crime convictions	0.5	(178/ 33,775)	**1.49**	(1.25, 1.78)	3.6	(1227/ 33,775)	**1.21**	(1.14, 1.29)

Note:

Bolded figures are significant at *p* < 0.05

Adjusted for year of birth, paternal age, maternal age, parity, maternal and paternal education, maternal conviction status

*Excluded 431,717 infants of non-violent fathers from the total study population

### Maternal half-sibling analyses

The findings for reoffending fathers and shortened gestational age remained (β − 0.91 [− 1.51 to − 0.31]) when comparing infants of reoffending fathers to his/her maternal half-sibling(s) fathered by men with no criminal conviction (Table 3). However, infants of fathers with 1–2 violent crime convictions did not have shortened gestational age compared to their maternal half-siblings (β 0.10 [− 0.36 to 0.56]). Corresponding comparisons for preterm birth appeared consistent with these results; however, limited by lowered statistical power from further restricted samples (OR_≥3_ 1.27 [0.91 to 1.78], OR _1–2_ 0.93 [0.71 to 1.21]).

### Sensitivity analysis

Associations between paternal violent criminality and preterm birth were more pronounced among births to cohabiting parents in comparison to non-cohabiting ones. Test of multiplicative interactions between paternal violent criminal conviction status (1–2 and 3 or more vs. no criminal conviction) and family situation on the odds ratio scale showed *p* value< 0.001. (Supplementary Table 3).

## Discussion

In this national cohort study of 1.5 million births, having a father convicted of violent crime, and especially a father who was repeatedly convicted, was associated with increased risk of preterm birth or shortened gestational age. The association was particularly pronounced for spontaneous preterm birth before 32 gestational weeks. The association between repeated paternal violent crime and shorter gestational age was confirmed when controlling for all invariant maternal characteristics shared between half-siblings.

Partner conviction status, used as a novel social indicator, may affect preterm birth risk in several ways. Explanations of the association include, but is not limited to, the mother herself being a victim of physical intimate partner violence [12–14]. Having a violent offender partner substantially increases the risk of reactive or proactive aggressive conflict solving. The higher risk in cohabiting couples indicates that paternal conviction may be a good indicator for stressful daily living situation of the mother. Such daily aggressive interactions may increase pregnant women’s psychological stress, and heighten the risk of preterm birth, especially spontaneous preterm birth through stress hormones [4, 17].

Paternal psychiatric illness cannot easily be disentangled from violent crime [18, 19]. In Sweden, offenders suffering even from severe mental disorders are not exempt from criminal trials and convictions. Therefore, paternal psychiatric disorder is likely to explain at least part of the association between paternal violent crime and preterm birth [20]. Men with more persistent antisocial behaviour are more likely to fulfil diagnostic criteria for personality disorders, ADHD, and substance misuse than those who had a short-term episode [18, 19]. Our robust finding of an association between repeated violent criminal offending and preterm birth also suggests that paternal mental health problems may mediate the association between violent offending and preterm birth. Nonetheless, not every convicted male has psychiatric morbidity, and social factors associated with conviction status could affect the mother in various ways.

Previous studies showed that conviction status may restrict the social and economic potential for both the offender himself and the remaining family [21, 22]. Previous studies on the impact of lower paternal social position on maternal and child health mostly focused incarceration [11, 23, 24]. Our findings, focusing on the type and frequency of convictions, provides additional support of criminal offender status as a social determinant of health [11].

Comparing pregnancies of the same mother allowed us to control for maternal invariant characteristics such as health risk behaviour and psychiatric morbidity that might correlate with the father’s conviction status. Analyses of exposure-discordant maternal half-siblings were hampered by statistical power, but did not essentially change the associations between paternal violent criminality and preterm birth. This further underscores the possible influence of a father with repeated violent convictions on the physical and psychosocial environment of the mother and the foetal environment.

### Strengths and limitations

The national register design of this study minimized selection and recall bias, which are major problems in previous studies of violence and preterm birth. The large sample size provided opportunities for analysing preterm birth subtypes, which might be more aetiologically informative. We also performed sibling comparisons which controlled for fixed maternal factors (i.e. factors not changing between pregnancies).

Although we used sibling analysis to further control for unmeasured maternal factors shared between siblings, confounders which vary between siblings may lead to more biased results [25]. We further adjusted for socioeconomic and behavioural factors that varied between siblings to reduce possible bias. Although there can be residual confounders that could possibly explain the associations, the consistency between the two analyses with adjustment for a wide range of measured (and unmeasured) confounders support a causal interpretation of the association.

Paternal violent crime conviction status is not only a strong proxy for paternal violent behaviour. All men who commit violent crimes are not reported, apprehended, prosecuted or convicted, and many of the crimes included in our indicator could be related to specific situations outside the family sphere. Thus, our findings are not generalizable to all intimate partner violence, the effect of which requires more precise information on partner relationship.

### Implications

This large national register study suggested that criminal behaviour or violence, even when not strictly measured as intimate partner violence, is a risk factor for preterm birth. The association between persistent paternal violent crime and preterm birth, especially very preterm birth, suggests that paternal violent and antisocial behaviour might affect the next generation even before birth. Future estimation of the public health repercussions of violence need to take preterm birth and its long-term health consequences into account.

It is important that health care professionals are aware of the possible link between paternal violence, maternal stress and preterm birth. Antenatal care consists of 10–12 visits to a midwife and is offered free of charge with 99% coverage in Sweden [26]. Screening questions regarding intimate partner violence are supposed to be routinely asked when alone with the woman. However, less than 80% of the women were actually asked the violence question during 2013–2014 according to the Swedish Pregnancy Register (Graviditetsregistret) that covers data from Stockholm and Gotland regions [27]. In Sweden, prospective fathers are encouraged to participate in antenatal care visits, parenthood education, and the labour process, which also provides chances to detect signs of violence, including threats. Since health professionals are at the frontier of detecting intimate partner violence, they should also be aware of resources for domestic violence services in the community and motivate or refer the patient when necessary. In addition, persistent reoffenders are more likely to suffer from psychiatric problems that require attention from health care professionals [18]. Integrated efforts from judicial, health care and social service authorities may benefit family members of persistent reoffenders.

## Conclusion

Paternal violent criminal conviction status is associated with increased risk of preterm birth. Half-siblings without a violent father have lower preterm birth risk than their half-sibling(s) with a convicted father. The association between paternal conviction status and risk of preterm birth deserves more attention from antenatal care providers and public health workers. Risk factors of expectant fathers requires further examination, especially regarding violent behaviour, poor mental health, and adverse social conditions.

## Supplementary information

**Additional file 1: Table S1.** Definition of violent crime according to the Swedish Penal Code (Ds 1999:36). **Table S2.** Conviction status of the father and odds ratios (ORs) of spontaneous/medically indicated very (≤31 weeks) preterm birth and moderately (32–36 weeks) preterm birth (*N* = 1,478,703). **Table S3.** Modification of family situation on paternal violent criminality status (*N* = 1,046,986*).

## Data Availability

The National Board of Health and Welfare in Sweden approved the request and provided the anonymous data after the ethical approval was obtained from the Regional Ethics Review Board. The data can be requested from the National Board of Health and Welfare in Sweden (https://www.socialstyrelsen.se/). The raw data cannot be shared in a public repository based on data use agreement.

## Abbreviations

aOR: Adjusted odds ratio
b: Mean gestational age in days
BMI: Body mass index
CI: Confidence interval
OR: Odds ratio
SMBR: Swedish Medical Birth Register
