# Supplementary material for: Paternal violent criminality and preterm birth: a Swedish national cohort study
Source: BMC Pregnancy Childbirth. 2020 May 19;20:307. doi: 10.1186/s12884-020-02964-2 (PMC7238610; doi:10.1186/s12884-020-02964-2)
Supplement: Supplementary file 1 — Additional file 1: Table S1. Definition of violent crime according to the Swedish Penal Code (Ds 1999:36). Table S2. Conviction status of the father and odds ratios (ORs) of spontaneous/medically indicated very (≤31 weeks) preterm birth and moderately (32–36 weeks) preterm birth (N = 1,478,703). Table S3. Modification of family situation on paternal violent criminality status (N = 1,046,986*). [file 12884_2020_2964_MOESM1_ESM.docx]

Supplementary Table 1. Definition of violent crime according to the Swedish Penal Code (Ds 1999:36).

| **Violent crime** | |  |  |
| --- | --- | --- | --- |
|  | Chapter 3 | Crimes against Life and Health | Section 1, 2, 3, 5, 6 |
|  | Chapter 4 | Crimes against Liberty and Peace | Section 1, 2, 4, 4a, 5, 7 |
|  | Chapter 6 | Sexual Crimes | Section 1-10, 12 |
|  | Chapter 8 | Theft, Robbery, and Other Crimes of Stealing | Section 5, 6 |
|  | Chapter 13 | Crimes Involving Public Danger | Section 1, 2 |
|  | Chapter 17 | Crimes Against Public Activity | Section 1 |
| **Non-Violent crime** | | The rest of the Swedish Penal Code | |

Supplementary Table 2. Conviction status of the father and odds ratios (ORs) of spontaneous/medically indicated very (≤31 weeks) preterm birth and moderately (32-36 weeks) preterm birth (N=1 478 703).

|  | Very preterm birth | | | |  | Moderately preterm birth | | | |
| --- | --- | --- | --- | --- | --- | --- | --- | --- | --- |
|  | % | (n/N) | OR | (95%CI) |  | % | (n/N) | OR | (95%CI) |
|  | **Medically indicated preterm birth** | | | |  |  |  |  |  |
| No Criminal Conviction | 0.2 | (2149/931630) | 1 |  |  | 1.2 | (10959/931630) | 1 |  |
| Convicted of Non-violent Crime | 0.2 | (1047/431717) | 1.01 | (0.93,1.09) |  | 1.2 | (5341/431717) | 1.01 | (0.97,1.04) |
| Convicted of Violent Crime | 0.3 | (308/115356) | 1.04 | (0.92,1.19) |  | 1.4 | (1590/115356) | 1.05 | (0.99,1.11) |
|  | **Spontaneous preterm birth** | | | |  |  |  |  |  |
| No Criminal Conviction | 0.3 | (2488/931630) | 1 |  |  | 2.7 | (24674/931630) | 1 |  |
| Convicted of Non-violent Crime | 0.3 | (1277/431717) | 1.03 | (0.96,1.11) |  | 2.8 | (12058/431717) | 1.02 | (1.00,1.05) |
| Convicted of Violent Crime | 0.4 | (471/115356) | **1.25** | (1.12,1.39) |  | 3.3 | (3780/115356) | **1.12** | (1.08,1.19) |

Note: Bolded figures are significant at p<0.05.

Adjusted for year of birth, paternal age, maternal age, parity, maternal and paternal education, maternal conviction status.

Supplementary Table 3. Modification of family situation on paternal violent criminality status (N=1 046 986*).

|  |  | **No crime conviction** | |  |  |  | **1-2 convicted violent crimes** | | |  |  | **3+convicted violent crimes** | | |
| --- | --- | --- | --- | --- | --- | --- | --- | --- | --- | --- | --- | --- | --- | --- |
|  | % | (n/N) | OR | (95%CI) |  | % | n/N | OR | (95%CI) |  | % | n | OR | (95%CI) |
| Living together | 4.33 | (37,242/ 860,329) | 1 |  |  | 5.01 | (3487/ 69,588) | **1.08** | (1.05, 1.13) |  | 5.92 | (1512/ 25,539) | **1.24** | (1.17, 1.31) |
| Not living together | 6.38 | (4552/ 71,301) | **1.40** | (1.36, 1.45) |  | 6.90 | (827/ 11,993) | **1.37** | (1.27, 1.47) |  | 7.58 | (624/ 8236) | **1.46** | (1.34, 1.59) |
|  |  |  |  |  |  |  |  |  |  |  |  |  |  |  |
|  |  | **ORs (95% CI) for paternal violent criminality within strata of cohabitation** | | | | | | | | | | | | |
| Living together |  |  | 1 |  |  |  |  | **1.07** | (1.03, 1.12) |  |  |  | **1.21** | (1.14, 1.29) |
| Not living together |  |  | 1 |  |  |  |  | 1.03 | (0.95, 1.12) |  |  |  | **1.12** | (1.01, 1.24) |

Note: Adjusted for year of birth, paternal age, maternal age, parity, maternal and paternal education, maternal conviction status.

*Excluded 431 717 infants of non-violent fathers from the total study population.
